# Construction of the gene regulatory network identifies MYC as a transcriptional regulator of SWI/SNF complex

**DOI:** 10.1038/s41598-019-56844-7

**Published:** 2020-01-13

**Authors:** Srimari Srikanth, Srimathy Ramachandran, Suma Mohan S

**Affiliations:** School of Chemical & Biotechnology, SASTRA Deemed to be University, Tirumalaisamudram, Thanjavur India

**Keywords:** Cancer genomics, Data integration

## Abstract

Precise positioning of nucleosomes at the gene regulatory elements mediated by the SWI/SNF family of remodelling complex is important for the transcriptional regulation of genes. A wide set of genes are either positively or negatively regulated by SWI/SNF. In higher eukaryotes, around thirty genes were found to code for SWI/SNF subunits. The construction of a gene regulatory network of SWI/SNF subunits identifies MYC as a common regulator for many of the SWI/SNF subunit genes. A meta-analysis study was conducted to investigate the MYC dependent regulation of SWI/SNF remodelling complex. Subunit information and the promoter sequences of the subunit genes were used to find the canonical E-box motif and its variants. Detailed analysis of mouse and human ChIP-Seq at the SWI/SNF subunit loci indicates the presence of MYC binding peaks overlapping with E-boxes. The co-expression correlation and the differential expression analysis of wt vs. MYC perturbed MEFs indicate the MYC dependent regulation of some of the SWI/SNF subunits. The extension of the analysis was done on MYC proficient and MYC deficient embryonic fibroblast cell lines, TGR1 and HO15, and in one of the MYC amplified cancer types, Medulloblastoma. A transcriptional regulatory feedback loop between MYC and SWI/SNF could be a major factor contributing to the aggressiveness of MYC dependent cancers.

## Introduction

The transcriptional regulation of a gene at its regulatory elements is orchestrated by transcription factors, cofactors, non-coding RNAs, epigenetic modifiers and chromatin remodelling complexes^1,2^. The SWI/SNF family of remodelling complex is known for its role in establishing nucleosome occupancy at target gene regulatory elements, by mobilizing nucleosomes in an ATP dependent manner^3^. These complexes are recruited to specific gene loci via association with transcription factors^4,5^ or nuclear receptors^6^ and do not show any DNA-sequence specificity^7^. These large multi-subunit complexes are formed by the assembly of about twelve subunits of different types such as core, accessory, and signature^8^. These subunits show tissue-specific expression pattern and can form different SWI/SNF complexes which perform specific functions in different tissues^9^. Importantly, the mutations of SWI/SNF subunits are highly prevalent in different cancer types and developmental disorders^10^. There are plenty of studies addressing the transcriptional regulatory role of SWI/SNF. However, there is a gap in our understanding of the regulation of SWI/SNF subunits and their role in contributing to the tissue-specific transcriptional plasticity.

In this study, we have identified MYC as a novel transcription regulator for different SWI/SNF subunits. MYC is a global transcription regulator and is one of the most well-studied proteins identified to be frequently deregulated in different human malignancies. The *MYC* oncogene was discovered by Bishop *et al*. in 1982, which encodes a helix-loop-helix leucine zipper protein^11,12^. Along with its functional partner MAX, MYC binds to the E-box sequence motif CAC(G/A)TG and its variants and mediate the transcriptional regulation of its target genes. Studies highlight the important role of MYC in embryonic development, cell growth, brain development and it plays a central role in regulating cell proliferation. Alterations in the *MYC* gene occur through chromosomal translocations and point mutations. The amplification of the *MYC* gene was observed in various human cancers, including breast carcinoma, cervical carcinoma, ovarian carcinoma, lung carcinoma, colon carcinoma and medulloblastoma^13^. As a transcription factor, MYC regulates a wide range of genes coding for proteins, miRs and lncRNAs and thereby coordinates key processes in cancer^14^. MYC plays the central role in oncogenesis by regulating proliferation, metabolism, differentiation and apoptosis. Targeting MYC is found to be a novel strategy in defeating such cancers. Hence it is of paramount importance to understand the functional regulators of MYC and its targets and their interplay in contributing to the aggressiveness of MYC dependent cancers.

Here, we have performed a meta-analysis study to understand the MYC dependent regulation of the SWI/SNF complex. Such meta-analysis approaches have been widely used to get insights into gene regulation mechanisms and disease associations based on publicly available datasets. For example, meta-analysis was employed to identify the direct targets of KAN1 transcription factor^15^, to find the shared biomarkers between thrombosis and myeloproliferative disorders^16^, to clarify the role of *SMARCA4* and *SMARCA2* in cancer^17^ and to identify the correlation between regulatory elements and gene expression in different cell types^18^. We have identified a role for MYC in regulating the subunits of SWI/SNF in fibroblast cells and Medulloblastoma. Apart from that, the co-expression correlation of MYC with different subunits of SWI/SNF observed in various cancer types available with the TCGA’s (The Cancer Genome Atlas), PanCancer Atlas. Previous studies have shown the regulation of *MYC* gene by SWI/SNF and interaction between MYC and SWI/SNF complex for the recruitment of SWI/SNF to the MYC target gene locus^19–22^. Our finding reveals a complex interplay between SWI/SNF and MYC in different cell types and may be an important factor to be considered for addressing the aggressiveness of MYC dependent cancers.

## Results

### Transcription regulatory network of SWI/SNF complex

**Figure 1:**
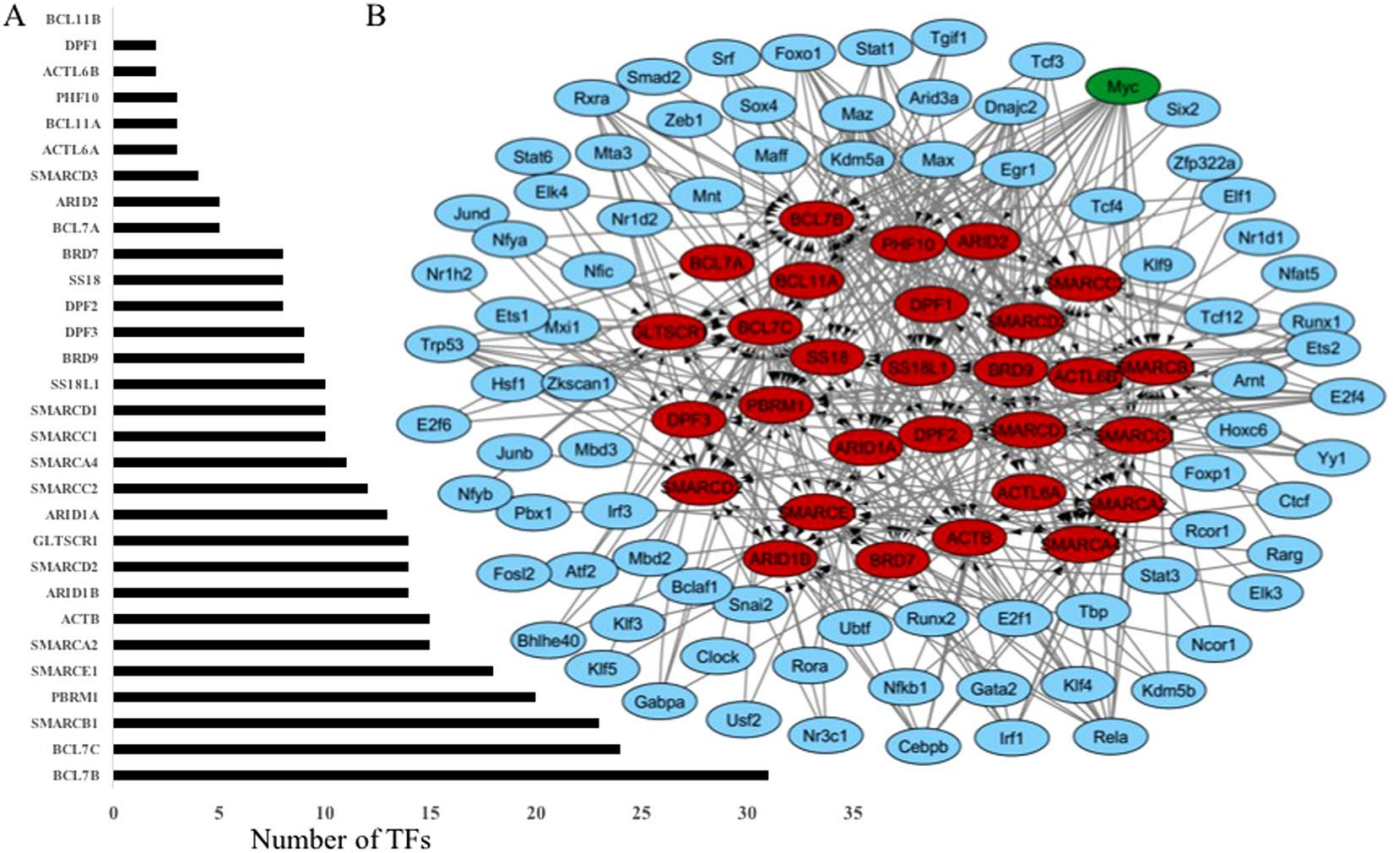
Gene regulatory network (GRN) of the SWI/SNF complex in MEF. (**A**) Number of TFs identified for each SWI/SNF Subunits using ChIPBase v2.0 (**B**) A gene regulatory network(GRN) of SWI/SNF Subunits in MEF constructed using Cytoscape.

To understand the regulatory mechanism of the SWI/SNF complex, the transcriptional regulatory network of SWI/SNF subunits was constructed based on Mouse Embryonic Fibroblasts(MEF). The details of SWI/SNF subunits in human and mouse used in the study and their chromosomal locations are given in supplementary table 1. The transcription factors of each SWI/SNF subunit in mouse were identified using ChIPBase v2.0, which uses ChIP-Seq data. Based on the expression status of these TFs in MEF, eighty-three TFs were used for the transcription regulatory network construction. The number of transcription factors identified for each SWI/SNF subunit is reported in Fig. 1A and the gene regulatory network(GRN) of SWI/SNF in MEF is visualized in Fig. 1B. The SWI/SNF subunits (represented in red color oval representation) and the TFs of each subunit are the nodes (blue color ovals) and the transcription regulation of the subunit by TFs are the edges in the directed graph representing the GRN of SWI/SNF complex. The GRN indicates that the TFs such as MYC, MAZ, RELA and EGR1 are involved in the regulation of 23, 15, 13, and 13 SWI/SNF genes, respectively. The proto-oncogene MYC is identified as the hub TF, which is possible to regulate the expression of twenty-three subunits of SWI/SNF (Fig. 1B).

### **E-box motifs and MYC binding peaks at the promoters of SWI/SNF subunit genes**

**Figure 2:**
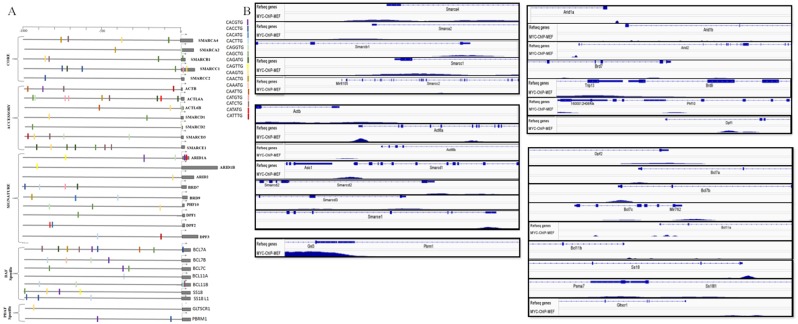
Myc binding sites in SWI/SNF subunit genes. (**A**) Presence of E-box motif and its variants in the regulatory region of SWI/SNF subunits in mouse (-1000-100 based on TSS. (**B**) MYC binding site in the promoters of SWI/SNF subunits from ChIP-Seq data in MEF.

The observed global regulatory role of MYC on the SWI/SNF complex was interesting to us and we further explored the possibility of Myc dependent regulation of SWI/SNF complex. It is known that MYC binds to the canonical E-box motif, “CACGTG” and its variants (CANNTG)^23^. To know whether the SWI/SNF subunit genes in mouse have the canonical E-box motif and their variants in their promoters, -1000 to 100 bp region based on the first exon start position of all the 30 SWI/SNF subunit genes were scanned for the presence of E-box motifs and its variants. The 1100 bp promoter sequence for the E-box motif analysis was obtained from the UCSC genome browser based on mouse genome assembly mm10. Using the FIMO algorithm in the MEME suite, the position of the E-box motif and its variants were marked in the promoter region of the SWI/SNF target genes. The E-box motif variants were marked with different colour codes, as shown in Fig. 2A. We could notice multiple occurrences of E-box motifs and its variants in most of the mouse SWI/SNF subunit gene promoters. The genes, *Smarcc1*, *Actl6a, Smarce1* and *Bcl7a* found to have the maximum number of E-box motifs. The genes such as *Smarca4, Smarca2, Smarcb1, Smarcc1, Actb, Actl6a, Actl6b, Smarcd2, Arid1a, Arid2, Bcl7a, Bcl11b* and *Pbrm1* contains E-box motifs within the 100 bp from the TSS. Only one E-box motif is present in genes such as *Smarcc2, Arid1b, Arid2, Dpf1, Bcl11a* and *Bicra*/*Gltscr1*. We extended the E-box motif analysis to human SWI/SNF subunit genes (based on genome assembly hg38). In human, we could notice the presence of E-box motif and its variants in all subunits except *ACTL6A* and *BRD7* (Supplementary figure 1A). The position and type of E-box motifs in the promoter of each subunit were found to vary between human and mouse.

Next, we wanted to know whether the E-box motifs identified in the promoter region of SWI/SNF subunits can be occupied by MYC. For that, we obtained the Genome-wide MYC binding profiles in different tissue types in human and mouse using the TFmapper database. The SWI/SNF subunit genes which have MYC binding across various tissues in mouse and human are tabulated in supplementary table 2 and 3 respectively. In mouse, the embryonic cell type has shown the maximum number of subunits with MYC binding possibility with twenty-five subunits, whereas in humans, blood, lung and mammary-gland cells list the maximum number of subunits genes regulated by MYC. The MYC binding peaks at the SWI/SNF gene promoter locus in MEF (GEO ID: GSE109458) were visualized using the Integrated genome viewer (IGV) by giving each gene’s corresponding chromosomal location by using mouse mm9 genome assembly (Fig. 2B). We could notice MYC binding peaks in MEF near the TSS of the SWI/SNF subunit genes such as *Smarca4, Smarca2, Smarcc1, Smarcd1, Actl6a, Arid1b, Arid2, Bcl7a, Bcl7b, Bcl11a, Brd9* and *Pbrm1*. Whereas the genes, *Smarcc2, Actl6b, Brd7, Dpf1, Phf10* and *Bicra/Gltscr1*, do not show MYC binding peaks. The MYC binding peaks overlapping with the E-box motifs and its variants were observed in all SWI/SNF subunits except *Smarcc2, Actl6b, Arid1b, Brd7, Phf10, Dpf1, Dpf2, Bcl11a* and *Bicra* (Supplementary table 4). The extension of the analysis in human based on a normal breast epithelial cell line, MCF10A (GEO ID: GSE31477) indicate the MYC binding at the promoters of *SMARCA4, SMARCA2, SMARCB1, SMARCC1, SMARCC2, ACTB, ACTL6A, SMARCD1, SMARCD2, SMARCD3, SMARCE1, ARID1A, ARID1B, ARID2, BRD7, BRD9, PHF10, DPF1, DPF2, DPF3, BCL7A, BCL7B, BCL7C, BCL11A, BCL11B, SS18, SS18L1, PBRM1* and *BICRA* genes (Supplementary figure 1B). The gene *ACTL6B* does not have a MYC binding peak in the MCF10A cell line.

### **Co-expression of SWI/SNF subunit genes with*MYC***

**Figure 3:**
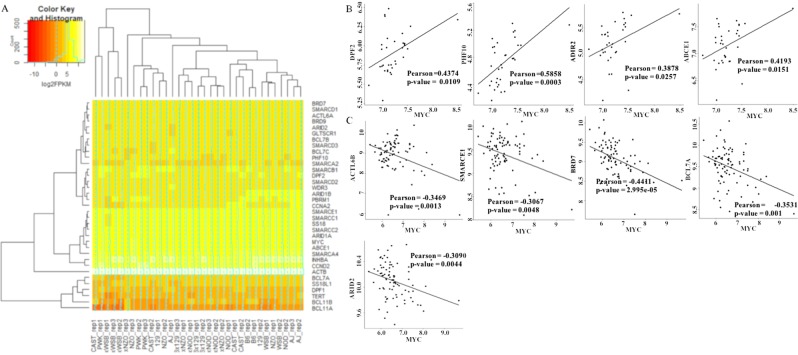
Gene expression analysis. (**A**) The heatmap for log2(FPKM) values of SWI/SNF subunit genes, *Myc* and MYC target genes in MEF. Co-expression correlation of SWI/SNF subunits with MYC in (**B**) MEF and (**C**) Medulloblastoma. Subunits showing significant co-expression correlation are displayed.

Next, we checked the expression levels of SWI/SNF subunits in MEFs. For that, we used the gene expression profiling experiment with GEO ID: GSE63756, which includes the RNA sequencing data of embryonic fibroblast samples from different strains of mouse. Using the control samples available in the experiment, the gene expression heat map was constructed for thirty SWI/SNF subunits and the MYC. The heat map shows the expression of the subunits of SWI/SNF in mouse embryonic fibroblast cells. The subunits *Bcl11a* and *Bcl11b* found to have low expression levels (Fig. 3A). In this study, we have included the known MYC target genes, *Abce1, Wdr3* as control.

The co-expression correlation of the SWI/SNF subunits with MYC was analysed based on the expression data from embryonic fibroblasts of different mouse strains used in the heatmap shown in Fig. 3A. The Pearson correlation coefficients obtained were 0.4374, 0.5858 and 0.3878 with, significant p-values for the subunits *Dpf2*, *Phf10* and *Arid2*, respectively. For the known MYC target gene, *Abce1*, the correlation coefficient was found to be 0.4193 (Fig. 3B). We observed MYC binding peaks in the promoter region of *Dpf2* and *Arid2* in MEF (Fig. 2B). The absence of MYC binding peak in *Phf10* may indicate the correlation between the subunits by an indirect mechanism mediated by MYC. Further, we have extended the co-expression correlation analysis to one of the MYC amplified cancer, Medulloblastoma (GEO ID: GSE50765). In that, we noticed a negative correlation of the subunits, *ACTL6B*, *SMARCE1*, *BRD7*, *BCL7A* and *ARID2* with MYC expression (Fig. 3C). Importantly, the correlation coefficient of the subunit *ARID2* in Medulloblastoma was found to be reversed as that in MEF and indicates the different ways of MYC dependent regulation possibilities on a gene. The known MYC target gene, *ABCE1*, doesn’t show any significant co-expression correlation in Medulloblastoma. We couldn’t obtain any publicly available ChIP-Seq data of MYC, to check the MYC dependent regulation possibility in Medulloblastoma.

We extended the co-expression correlation analysis of *MYC* with SWI/SNF subunits to an extended list of cancers available in the TCGA’s PanCancerAtlas through cBioPortal and the correlation details are summarized in supplementary table 5. The Pearson correlation coefficient for SWI/SNF subunit genes with *MYC* in each cancer types is tabulated in the table. The SWI/SNF subunit genes showing the Pearson correlation coefficient >= 3 are highlighted in each cancer type. The cancer types Acute Myeloid Leukemia (with 11 subunits showing correlation), Thymoma (10), and Uveal Melanoma (10) are the ones with most subunits showing co-expression correlation with *MYC*. Whereas in Liver Hepatocellular Carcinoma, Lung Adenocarcinoma and Ovarian Serous Cystadenocarcinoma, we could not observe any significant correlation of SWI/SNF subunits with *MYC*. The genes, *SMARCC1, ACTB, SMARCD3, BCL11A, ACTL6A and BCL7A* found to have a correlated expression with *MYC* in more than five cancer types reported in supplementary table 5.

### **Differential Expression analysis of SWI/SNF subunits in MYC perturbed conditions**

**Figure 4:**
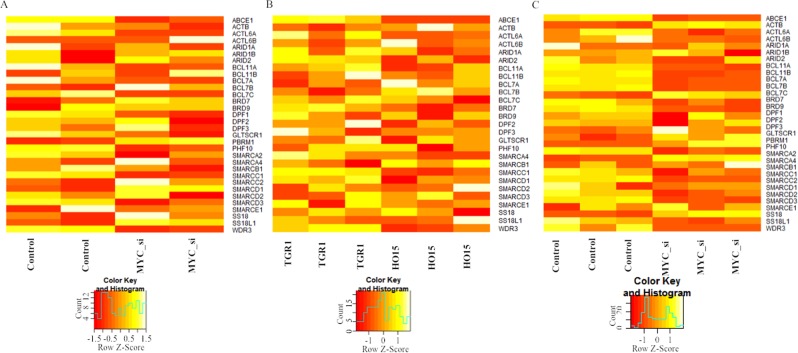
(**A**) Heatmap showing expression patterns of the SWI/SNF subunit genes in control and Myc siRNA treated MEF cell line (**B**)TGR1 and HO15 (**C**)Medulloblastoma.

Since there was a co-expression correlation between some of the SWI/SNF subunits and *Myc* in MEF, we went on to check whether the subunits show any MYC dependent expression pattern. For that, we obtained the gene expression profiles in MYC perturbed conditions in MEF. The gene expression profile by RNA-Seq in MEF with GEO ID: GSE67715, which contains two experimental replicates for control and *Myc* silenced conditions^24^, was considered for the differential expression analysis. The heat map showing expression patterns of the SWI/SNF subunit genes in control and c-Myc siRNA treated MEF cell line is shown in Fig. 4A. The results from the differential expression analysis in wt vs. Myc si-RNA treated MEF is reported in supplementary table 6. From this data set, none of the SWI/SNF subunits turned up with significant adj.P-value. The microarray-based expression profiling in MEF in normal vs. Myc silenced without replicates show significant differential expression for all the subunits (Supplementary Table 6).

**Figure 5:**
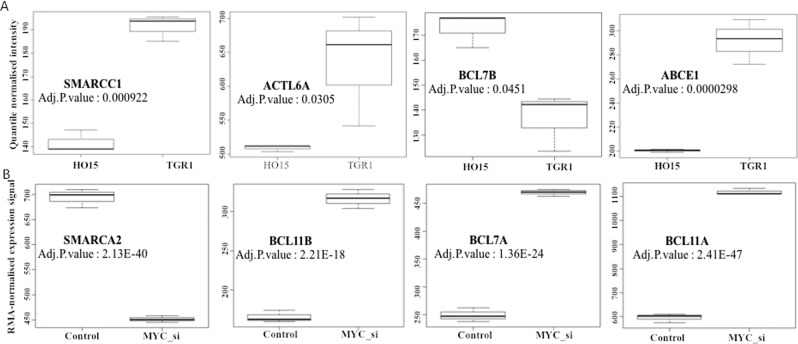
(**A**) Boxplot showing significantly differentially expressed genes TGR1 vs. HO15 (**B**) Differentially expressed genes in MYC perturbed conditions in Medulloblastoma (Only top four SWI/SNF subunits are shown).

We extended the differential expression analysis to *Myc* proficient and deficient rat fibroblast cell lines, TGR1 and HO15, respectively. The gene expression data from RNA-Seq used here was obtained from the GEO profile with GEO ID: GSE18845, which contains three experimental replicates for TGR1 and HO15^25^. Then for the specific subunit genes showing significant differential expression in TGR1 vs. HO15 (Supplementary Table 5) were represented as Boxplots. The boxplots for the genes *Smarcc1*, *Actl6a* and *Bcl7b* showing significant differential expression in Myc proficient and deficient conditions are shown in Fig. 5A.

We further extended the analysis to one of the MYC proficient cancer cell models, Medulloblastoma (Fig. 4C) where we observed differential expression of most of the SWI/SNF subunits with significant adj.P-values. Differential gene expression analysis was done in control vs. *MYC* silenced conditions in MYC overexpressing clones where expression details of three replicates were present (GEO ID: GSE22139). The differential expression analysis and heatmap (Supplementary Table 6 and Fig. 4C) indicate differential expression in wt vs. *MYC* perturbed conditions in subunits such as *SMARCA2, BCL11B, BCL7A, BCL11A, SS18, SMARCC1, SMARCD3, BCL7C, BCL7B* and *PBRM1* with significant adj.P-value (Fig. 5B, Supplementary Table 6). Seitz *et al*. reports the MYC dependent differential expression of *SMARCC1* subunit gene and MYC binding in different SWI/SNF subunit locus in another MYC amplified cancer type, Burkitt Lymphoma^26^ whereas the study reported in Lung adenocarcinoma do not report the MYC dependent expression any of the SWI/SNF subunits^27^.

## Discussion

The transcriptional regulation mediated by the SWI/SNF family of remodelling complex is an active area of research and is known to control the gene expression programs in eukaryotes starting from the embryogenesis through cell differentiation and development into various tissues^28^. However, the regulation of the SWI/SNF complex, the formation of tissue-specific sub-complexes and its role in controlling tissue-specific gene expression programs are not widely explored so far. To understand the transcriptional regulatory programs of SWI/SNF, we have constructed a gene regulatory network of SWI/SNF subunits in MEF and observed a connection between the well-known proto-oncogene MYC and the SWI/SNF family of remodelling complex(Fig. 1B).

**Figure 6:**
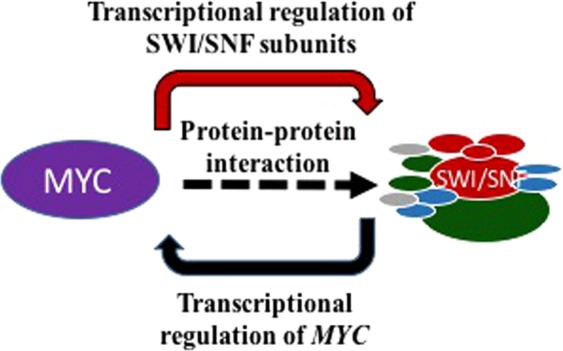
Multi-level connections between MYC and SWI/SNF.

Being a global transcriptional regulator, several key genes involved in differentiation, cell cycle, cell growth, proliferation, metabolism and ribosomal biogenesis are known to be controlled by the proto-oncogene MYC^29^. The functional connection between MYC and SWI/SNF is already established at different levels. (1) The MYC protein is known to interact with the SWI/SNF subunit BAF47*(SMARCB1)* (5, 21) and with this interaction, (2) Myc recruits the SWI/SNF complex to its target gene locus for the transcriptional regulation^30^. (3) Importantly, the SWI/SNF complex is known to regulate *MYC* transcriptionally. Nagl Jr. *et al*. reported a direct regulation of the *MYC* gene by the promoter binding activity of SWI/SNF. The *ARID1A* subunit of SWI/SNF, which is required for the differentiation-associated cell cycle arrest and involved in targeting cell cycle-regulated genes, binds to the *MYC* promoter during differentiation^19^. Apart from that, there are reports on the enhancer-mediated *MYC* regulatory function of SWI/SNF in Acute Leukemia maintenance^20^. In aggressive B-Cell Lymphomas, the NFAT family of transcription factors are known to recruit SWI/SNF remodelling complex for the transcriptional regulation of *MYC* oncogene^31^. The ATPase subunit of SWI/SNF, BRG1, is known to regulate *MAX*, which is the important functional partner of MYC. Here, we provide evidence for the Myc dependent regulation of SWI/SNF subunits through a meta-analysis approach. The multilevel interaction between MYC and SWI/SNF is depicted in the schematic diagram given in Fig. 6.

In the present meta-analysis study, we obtained evidences for the MYC dependent regulation of SWI/SNF remodelling complex based on multiple inputs such as the presence of E-box motifs, genome-wide MYC binding profile from multiple cell types in human and mouse, co-expression correlation analysis of the gene expression dataset and differential expression in MYC perturbed conditions. The presence of E-box motifs in SWI/SNF subunits of human and mouse and MYC binding peaks at the promoter region of SWI/SNF subunits in MEF and MCF10A indicates the possibility of transcription control mediated by promoter binding activity of MYC (Fig. 2 and Supplementary figure 1). The co-expression correlation analysis indicates the positive correlation of the subunits, *Dpf2*, *Phf10* and *Arid2* in MEF and negative correlation of subunits, *ACTL6B*, *SMARCE1*, *BRD7*, *BCL7A* and *ARID2* with MYC in Medulloblastoma (Fig. 3B and C). The extended co-expression correlation analysis in different cancer types in TCGA’s PanCancerAtlas indicates the different possibilities of Myc dependent regulation of different SWI/SNF subunits in various cancer types (Supplementary Table 5). The summary of the SWI/SNF subunit regulation by MYC observed in the meta-analysis is reported in supplementary table 7. In the case of subunits such as *SMARCA4*, *ACTB*, *SMARCE1*, *ARID2*, *PHF10*, *BCL7A*, *SS18*, *SS18L1* and *BICRA*/*GLTSCR1*, we observed a dual role of MYC in the activation as well as repression (Supplementary Table 7). MYC is reported to elicit differential regulation at the target gene locus based on the association with other interacting factors^32^. The differential expression analysis in wt vs. MYC perturbed conditions reports the functional validation of the meta-analysis performed. From the differential expression analysis in rat fibroblasts, the down-regulation of *Smarcc1* and *Actl6a* and up-regulation of *Bcl7b* observed in HO15(MYC deficient) compared to the TGR1 (MYC proficient). In medulloblastoma, we noticed the downregulation of subunits such as *SMARCA2*, *ACTB*, *BCL7C*, *GLTSCR1/BICRA, SS18* and *PBRM1* and upregulation of *SMARCC1, SMARCD2, SMARCD3, BRD9, BCL7A, BCL7B, BCL11A* and *BCL11B* in MYC silenced conditions (Fig. 5 and Supplementary Table 6). However, we could not notice significant up/down regulation of the SWI/SNF subunits in one of the differential expression analysis in MEF based on GEO dataset GSE67715 reported in supplementary table 6. But MYC binding evidence are present in the promoter region of most of the SWI/SNF subunits in MEF (Fig. 2B). Therefore, our meta-analysis paves the way for further experimental research to unravel the interconnection between MYC and SWI/SNF complex at the transcriptional level. Importantly, consistent with the observations from the meta-analysis study, we could find functional evidence for the MYC dependent regulation of two of the SWI/SNF subunits, BRD7 and PHF10^33,34^. Liu Y *et al*. report the MYC mediated negative regulation of BRD7 subunit^33^ and is consistent with our co-expression correlation analysis reported BRD7 in Fig. 3C (Pearson correlation coefficient -0.4411). In another study, Tatarskiy *et al*. reported the positive regulation of the SWI/SNF subunit, PHF10 by MYC in the SW620 cell line^34^ as observed in the co-expression correlation analysis of PHF10 reported in MEF (Fig. 3B) (Pearson correlation coefficient 0.5858). However, the PHF10 subunit found to have a negative coexpression correlation in Head and Neck Squamous Cell Carcinoma (Supplementary Table 5). It is important to study the functional connection between other regulatory factors of the MYC and MYC family of proteins in mediating different ways of regulation in different tissue types. It has been reported that during differentiation and cell growth, the MYC family of proteins regulate the same set of target genes and can compensate for one another. Also, MYCN is known to replace MYC during murine development functionally^35^.

This is the first kind of a systematic study reported so far to identify transcriptional regulation of SWI/SNF subunits by MYC. The transcriptional regulation of SWI/SNF subunits by MYC is not explored so far apart from some isolated studies listing a few SWI/SNF subunits in the MYC target gene list and the recent reports on PHF10 and BRD7^33,34,36,37^. MYC is one of the most amplified proteins in different cancer types and the SWI/SNF remodeling complex is gaining importance for the frequency of cancer-associated inactivating mutations present in its subunits^29,38^. MYC and different members of the SWI/SNF family of proteins have been shown to be involved in regulating cell growth and proliferation. This may suggest that the MYC dependent proliferation programs might be utilizing the SWI/SNF family of remodeling complexes. Since both factors, SWI/SNF and MYC play an important role in the tissue-specific gene expression and cell proliferation^39,40^, understanding and exploring the functional interconnection between MYC and SWI/SNF is critical in tracking the aggressiveness of MYC dependent cancers. Romero OA *et al*. reports that the aberrant SWI/SNF-MYC network plays an important role in lung cancer development^22^. MYC is also known to mediate tissue-specific gene regulation by interacting with different functional partners and known to have different target lists in different tissues^36^. Therefore, exploring the functional connection between MYC and SWI/SNF can be considered as a major factor in understanding the role of SWI/SNF in controlling tissue-specific gene expression programs and in developing strategies for novel therapies for MYC dependent cancers.

## Methods

### **Construction of the gene regulatory network of SWI/SNF subunits in MEF**

The subunit details of human and mouse SWI/SNF complex were obtained from SWI/SNF Infobase^8^. The transcription factors(TFs) involved in the regulation of thirty SWI/SNF subunit genes in mouse were identified using ChIPBase v2.0^41^. Based on the expression statues in Mouse Embryonic Fibroblasts(MEF) (based on GEO profile, GSE46645), 83 out of 189 TFs were subjected to gene regulatory network construction using Cytoscape (version 3.7.0)^42^. The hub TFs in the network were identified based on the out-degree of nodes.

### **The promoter region of SWI/SNF subunit genes**

Promoter sequences for the thirty SWI/SNF subunit genes in human and mouse were obtained from the UCSC genome browser^43^ based on the genome assembly hg38 and mm10, respectively. The promoter region considered for analysis was 1100 bp region covering -1000 to 100 bp based on the first exon start site.

### **Identification of E-Box motifs**

The Canonical E-box motifs and its variants present in the promoter regions of human and mouse SWI/SNF subunit genes were identified using the sequence scanning algorithm FIMO (Find Individual Motif Occurrences) available with MEME suite^44^. The canonical E-box motif “CACGTG” and its variants^23^ based on the sequence pattern “CANNTG” (a total of sixteen motifs) were included in the analysis.

### **Analysis of Genome-wide MYC Binding profiles**

Genome-wide MYC binding details for human and mouse based on ChIP-Seq/DNase-Seq/ATAC-Seq were obtained from TFmapper^45^. Parameters used to obtain the MYC binding details are as follows: Species:Mouse(mm9), human(hg19), IP: Trans-acting factors, Database: GEO, Biological source: All, search by gene (thirty SWI/SNF subunit genes), Portion of the gene to query: Promoter. Binding details were obtained individually for each gene and the results were downloaded in table format.

**Figure 7:**
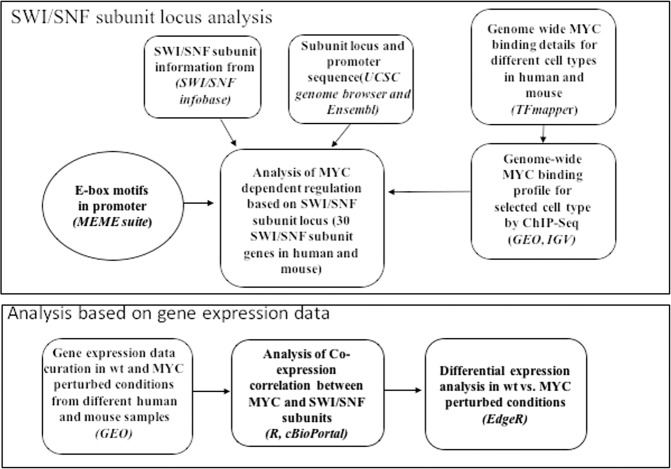
The Meta-analysis strategy used in the study.

The MYC binding profiles from ChIP-Seq experiments were obtained from GEO for the selected cell types (MEF in mouse and MCF10A in human) identified using the TFmapper. The Wiggle formatted files were obtained from GEO and the MYC binding peaks at the promoter regions of SWI/SNF subunits genes were visualized using IGV (version 2.4)^46^. The details of the gene expression datasets and the MYC binding profiles used in the study and the analysis details are reported in supplementary table 8 and the meta-analysis strategy used in the study is reported in Fig. 7.

### **Co-expression correlation analysis**

We have used the cBioPortal online platform to obtain the Pearson correlation coefficient for the co-expression of SWI/SNF subunit genes with MYC in different cancer models available with TCGA's PanCancerAtlas.

### **Differential gene expression analysis in MYC perturbed conditions**

To understand the MYC dependent expression of SWI/SNF subunits, the gene expression profiles of Myc perturbed conditions in MEF and different human cell types and the details were obtained and tabulated in supplementary table 6. The expression profiles were obtained from GEO in MYC perturbed conditions such as Silenced c-Myc, Minimal c-Myc, Myc siRNA treated, and Myc KD from available cell types and were subjected to differential expression analysis. Differential gene expression analysis of wt and Myc perturbed conditions in MEF, embryonic fibroblast from rat, TGR, HO15 and human medulloblastoma, cancer showing enhanced expression of MYC were done. The analysis was done using the edgeR package in R (version 3.4.3)^47^. The expression details were represented by heatmaps and boxplots using R. The co-expression analysis and the plots were generated using R^48^.

## Data availability

The data used for the present meta-analysis study is publicly available and the accession details of the data used are provided in the manuscript.

## Supplementary information

Supplementary Information.
