# Supplementary material for: Construction of the gene regulatory network identifies MYC as a transcriptional regulator of SWI/SNF complex
Source: Sci Rep. 2020 Jan 13;10:158. doi: 10.1038/s41598-019-56844-7 (PMC6957478; doi:10.1038/s41598-019-56844-7)
Supplement: Supplementary file 1 — Supplementary Information. [file 41598_2019_56844_MOESM1_ESM.docx]

**Construction of the gene regulatory network identifies MYC as a transcriptional regulator of SWI/SNF complex**

# Srimari Srikanth 1, Srimathy Ramachandran 1, and Suma Mohan S1,*

1School of Chemical & Biotechnology, SASTRA Deemed to be University, Tirumalaisamudram, Thanjavur, India.

*sumamohan@scbt.sastra.edu

**Supplementary tables and figures**

| **Sl. No.** | **Type of Subunit** | **Gene official symbol** | **Description** | **Protein Name** | **Chromosomal location in human** | **Chromosomal location in Mouse** |
| --- | --- | --- | --- | --- | --- | --- |
| 1 | Core | *SMARCA4* | SWI/SNF related, matrix associated, actin dependent regulator of chromatin, subfamily a, member 4 | Transcription activator BRG1 | 19p13.2 | 9qA3 |
| 2 |  | *SMARCA2* | SWI/SNF related, matrix associated, actin dependent regulator of chromatin, subfamily a, member 2 | Probable global transcription activator SNF2L2 | 9p24.3 | 19qC1 |
| 3 |  | *SMARCB1* | SWI/SNF related, matrix associated, actin dependent regulator of chromatin, subfamily b, member 1 | SWI/SNF-related matrix-associated actin-dependent regulator of chromatin subfamily B member 1 | 22q11.23 | 10qC1 |
| 4 |  | *SMARCC1* | SWI/SNF related, matrix associated, actin dependent regulator of chromatin subfamily c member 1 | SWI/SNF complex subunit SMARCC1 | 3p21.31 | 9qF2 |
| 5 |  | *SMARCC2* | SWI/SNF related, matrix associated, actin dependent regulator of chromatin subfamily c member 2 | SWI/SNF complex subunit SMARCC2 | 12q13.2 | 10qD3 |
| 6 | Accessory | *ACTB* | actin beta | Actin, cytoplasmic 1 | 7p22.1 | 5qG2 |
| 7 |  | *ACTL6A* | actin like 6A | **Actin-like protein 6A** | 3q26.33 | 3qA3 |
| 8 |  | *ACTL6B* | actin like 6B | **Actin-like protein 6B** | 7q22.1 | 5qG2 |
| 9 |  | *SMARCD1* | SWI/SNF related, matrix associated, actin dependent regulator of chromatin, subfamily d, member 1 | SWI/SNF-related matrix-associated actin-dependent regulator of chromatin subfamily D member 1 | 12q13.12 | 15qF1 |
| 10 |  | *SMARCD2* | SWI/SNF related, matrix associated, actin dependent regulator of chromatin, subfamily d, member 2 | SWI/SNF-related matrix-associated actin-dependent regulator of chromatin subfamily D member 2 | 17q23.3 | 11qE1 |
| 11 |  | *SMARCD3* | SWI/SNF related, matrix associated, actin dependent regulator of chromatin, subfamily d, member 3 | SWI/SNF-related matrix-associated actin-dependent regulator of chromatin subfamily D member 3 | 7q36.1 | 5qA3 |
| 12 |  | *SMARCE1* | SWI/SNF related, matrix associated, actin dependent regulator of chromatin, subfamily e, member 1 | SWI/SNF-related matrix-associated actin-dependent regulator of chromatin subfamily E member 1 | 17q21.2 | 11qD |
| 13 | Signature | *ARID1A* | AT-rich interaction domain 1A | AT-rich interactive domain-containing protein 1A | 1p36.11 | 4qD2.3 |
| 14 |  | *ARID1B* | AT-rich interaction domain 1B | AT-rich interactive domain-containing protein 1B | 6 q25.3 | 17qA1 |
| 15 |  | *ARID2* | AT-rich interaction domain 2 | AT-rich interactive domain-containing protein 2 | 12q12 | 15qF1 |
| 16 |  | *BRD7* | bromodomain containing 7 | Bromodomain-containing protein 7 | 16q12.1 | 8qC3 |
| 17 |  | *BRD9* | bromodomain containing 9 | Bromodomain-containing protein 9 | 5p15.33 | 13qC1 |
| 18 |  | *PHF10* | PHD finger protein 10 | PHD finger protein 10 | 6q27 | 17qA2 |
| 19 |  | *DPF1* | double PHD fingers 1 | Zinc finger protein neuro-d4 | 19q13.2 | 7qB1 |
| 20 |  | *DPF2* | double PHD fingers 2 | Zinc finger protein ubi-d4 | 11q13.1 | 19qA |
| 21 |  | *DPF3* | double PHD fingers 3 | Zinc finger protein DPF3 | 14q24.2 | 12qD1 |
| 22 | BAF specific | *BCL7A* | BAF chromatin remodelling complex subunit BCL7A | B-cell CLL/lymphoma 7 protein family member A | 12q24.31 | 5qF |
| 23 |  | *BCL7B* | BAF chromatin remodelling complex subunit BCL7B | B-cell CLL/lymphoma 7 protein family member B | 7q11.23 | 5qG2 |
| 24 |  | *BCL7C* | BAF chromatin remodelling complex subunit BCL7C | B-cell CLL/lymphoma 7 protein family member C | 16p11.2 | 7qF3 |
| 25 |  | *BCL11A* | BAF chromatin remodelling complex subunit BCL11A | B-cell lymphoma/leukaemia 11A | 2p16.1 | 11qA3.2 |
| 26 |  | *BCL11B* | BAF chromatin remodelling complex subunit BCL11B | B-cell lymphoma/leukaemia 11B | 14q32.2 | 12qF1 |
| 27 |  | *SS18* | SS18 subunit of BAF chromatin remodelling complex | Protein SSXT | 18q11.2 | 18qA1 |
| 28 |  | *SS18L1* | SS18L1 subunit of BAF chromatin remodelling complex | Calcium-responsive trans activator | 20q13.33 | 2qH4 |
| 29 |  | *GLTSCR1/BICRA* | BRD4 interacting chromatin remodelling complex associated protein | BRD4-interacting chromatin-remodelling complex-associated protein | 19q13.33 | 7qA2 |
| 30 | PBAF specific | *PBRM1* | polybromo 1 | Protein polybromo-1 | 3p21.1 | 14qB |

**Supplementary Table 1**: The details of SWI/SNF subunits in Human and Mouse

**
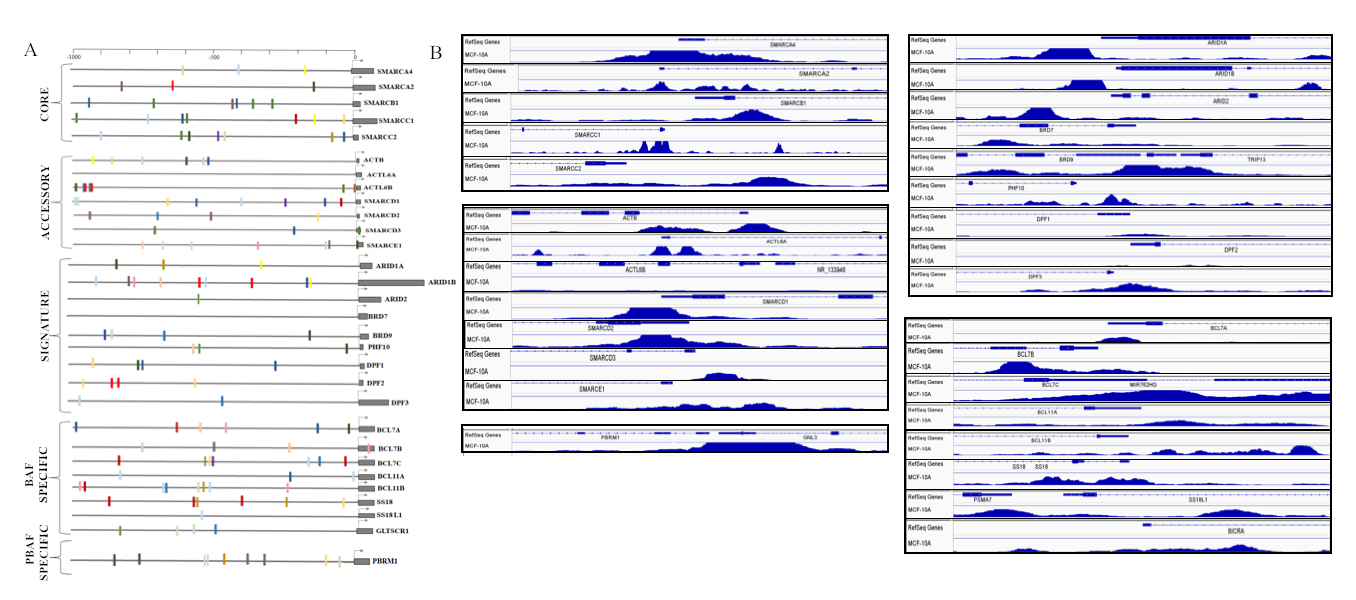
**

**Supplementary Figure 1: MYC binding sites in SWI/SNF subunit genes in human.** (A) Presence of E-box motif and its variants in the regulatory region of SWI/SNF subunits in mouse (-1000-100 based on TSS. (B) MYC binding sites in the promoters of SWI/SNF subunits from ChIP-Seq data in MCF10A

| **Subunit/Tissue** | **Breast** | **Umbilical Vein** | **Bone** | **Colon** | **Liver** | **Embryo** | **Skin** | **None** | **Brain** | **Cervix** | **Embryonic Kidney** | **Bone marrow** | **Blood** | **Lung** | **Mammary Gland** |
| --- | --- | --- | --- | --- | --- | --- | --- | --- | --- | --- | --- | --- | --- | --- | --- |
| **Core** | *SMARCA4* | *SMARCA4* | *SMARCA4* | *SMARCA4* | *SMARCA4* | *SMARCA4* | *SMARCA4* | *SMARCA4* | *SMARCA4* | *SMARCA4* | *SMARCA4* | *SMARCA4* | *SMARCA4* | *SMARCA4* | *SMARCA4* |
|  | *SMARCA2* | *SMARCA2* | *SMARCA2* | *SMARCA2* | *-* | *SMARCA2* | *SMARCA2* | *SMARCA2* | *SMARCA2* | *SMARCA2* | *SMARCA2* | *SMARCA2* | *SMARCA2* | *SMARCA2* | *SMARCA2* |
|  | *-* | *-* | *-* | *-* | *-* | *-* | *-* | *-* | *-* | *-* | *SMARCB1* | *-* | *-* | *-* | *-* |
|  | *SMARCC1* | *SMARCC1* | *SMARCC1* | *SMARCC1* | *SMARCC1* | *-* | *SMARCC1* | *SMARCC1* | *SMARCC1* | *SMARCC1* | *SMARCC1* | *SMARCC1* | *SMARCC1* | *SMARCC1* | *SMARCC1* |
|  | *SMARCC2* | *SMARCC2* | *SMARCC2* | *SMARCC2* | *SMARCC2* | *SMARCC2* | *SMARCC2* | *SMARCC2* | *SMARCC2* | *SMARCC2* | *SMARCC2* | *SMARCC2* | *SMARCC2* | *SMARCC2* | *SMARCC2* |
| **Accessory** | *-* | *-* | *ACTB* | *-* | *-* | *ACTB* | *-* | *ACTB* | *ACTB* | *ACTB* | *ACTB* | *ACTB* | *ACTB* | *ACTB* | *ACTB* |
|  | *-* | *-* | *-* | *ACTL6A* | *-* | *-* | *ACTL6A* | *-* | *ACTL6A* | *ACTL6A* | *ACTL6A* | *ACTL6A* | *ACTL6A* | *ACTL6A* | *ACTL6A* |
|  | *-* | *-* | *-* | *-* | *-* | *-* | *-* | *-* | *-* | *-* | *ACTL6B* | *ACTL6B* | *-* | *ACTL6B* | *-* |
|  | *SMARCD1* |  | *SMARCD1* | *SMARCD1* | *SMARCD1* | *SMARCD1* | *SMARCD1* | *SMARCD1* | *-* | *SMARCD1* | *SMARCD1* | *SMARCD1* | *SMARCD1* | *SMARCD1* | *SMARCD1* |
|  | *SMARCD2* | *SMARCD2* | *SMARCD2* | *SMARCD2* | *SMARCD2* | *SMARCD2* | *SMARCD2* | *SMARCD2* | *SMARCD2* | *SMARCD2* | *SMARCD2* | *SMARCD2* | *SMARCD2* | *SMARCD2* | *SMARCD2* |
|  | *-* | *-* | *SMARCD3* | *SMARCD3* | *-* | *SMARCD3* | *SMARCD3* | *-* | *SMARCD3* | *SMARCD3* | *SMARCD3* | *SMARCD3* | *SMARCD3* | *SMARCD3* | *SMARCD3* |
|  | *-* | *SMARCE1* | *SMARCE1* | *SMARCE1* | *SMARCE1* | *SMARCE1* | *-* | *-* | *SMARCE1* | *-* | *SMARCE1* | *SMARCE1* | *SMARCE1* | *SMARCE1* | *SMARCE1* |
| **Signature** | *ARID1A* | *ARID1A* | *ARID1A* | *ARID1A* | *ARID1A* | *ARID1A* | *ARID1A* | *ARID1A* | *ARID1A* | *ARID1A* | *ARID1A* | *ARID1A* | *ARID1A* | *ARID1A* | *ARID1A* |
|  |  | *ARID1B* | *ARID1B* | *-* | *ARID1B* | *ARID1B* |  | *ARID1B* | *ARID1B* | *ARID1B* | *ARID1B* | *ARID1B* | *ARID1B* | *ARID1B* | *ARID1B* |
|  | *ARID2* | *ARID2* | *ARID2* | *-* | *ARID2* | *ARID2* | *ARID2* | *ARID2* | *ARID2* | *ARID2* | *ARID2* | *ARID2* | *ARID2* | *ARID2* | *ARID2* |
|  |  |  | *BRD7* | *-* | *BRD7* | *BRD7* | *-* | *BRD7* | *BRD7* | *BRD7* | *-* | *BRD7* | *BRD7* | *BRD7* | *BRD7* |
|  | *BRD9* | *BRD9* | *BRD9* | *BRD9* | *BRD9* | *BRD9* | *BRD9* | *BRD9* | *BRD9* | *BRD9* | *BRD9* | *BRD9* | *BRD9* | *BRD9* | *BRD9* |
|  | *-* | *-* | *PHF10* | *-* | *PHF10* | *PHF10* | *PHF10* | *PHF10* | *PHF10* | *PHF10* | *PHF10* | *PHF10* | *PHF10* | *PHF10* | *PHF10* |
|  | *DPF1* | *-* | *-* | *DPF1* | *DPF1* | *DPF1* | *DPF1* | *DPF1* | *DPF1* | *DPF1* | *DPF1* | *DPF1* | *DPF1* | *DPF1* | *DPF1* |
|  | *-* | *-* | *-* | *-* | *-* | *-* | *DPF3* | *-* | *-* | *DPF3* | *-* | *DPF3* | *-* | *-* | *DPF3* |
|  | *-* | *DPF2* | *-* | *DPF2* | *-* | *-* | *-* | *DPF2* | *-* | *DPF2* | *DPF2* | *DPF2* | *DPF2* | *DPF2* | *DPF2* |
| **BAF Specific** | *-* | *BCL7A* | *-* | *-* | *-* | *-* | *BCL7A* | *-* | *-* | *BCL7A* | *BCL7A* | *BCL7A* | *BCL7A* | *BCL7A* | *BCL7A* |
|  | *-* | *BCL7B* | *BCL7B* | *BCL7B* | *BCL7B* | *BCL7B* | *BCL7B* | *BCL7B* | *BCL7B* | *BCL7B* | *BCL7B* | *BCL7B* | *BCL7B* | *BCL7B* | *BCL7B* |
|  | *BCL7C* | *BCL7C* | *BCL7C* | *BCL7C* | *BCL7C* | *BCL7C* | *BCL7C* | *BCL7C* | *-* | *BCL7C* | *BCL7C* | *BCL7C* | *BCL7C* | *BCL7C* | *BCL7C* |
|  | *-* | *-* | *-* | *-* | *-* | *-* | *-* | *-* | *-* | *-* | *-* | *-* | *BCL11A* | *BCL11A* | *BCL11A* |
|  | *-* | *BCL11B* | *-* | *BCL11B* | *-* | *-* | *BCL11B* | *-* | *BCL11B* | *-* | *BCL11B* | *-* | *BCL11B* | *BCL11B* | *BCL11B* |
|  | *-* | *SS18* | *-* | *SS18* | *SS18* | *-* | *-* | *SS18* | *SS18* | *SS18* | *SS18* | *SS18* | *SS18* | *SS18* | *SS18* |
|  | *-* | *-* | *-* | *-* | *SS18L1* | *-* | *-* | *-* | *-* | *-* | *-* | *SS18L1* | *SS18L1* | *SS18L1* | *SS18L1* |
| **PBAF Specific** | *-* | *PBRM1* | *-* | *-* | *-* | *-* | *-* | *PBRM1* | *-* | *PBRM1* | *PBRM1* | *PBRM1* | *PBRM1* | *PBRM1* | *PBRM1* |

**Supplementary table 2**: SWI/SNF subunit genes which have MYC binding peaks at the promoter regions identified using TFmapper across various tissues in mouse

| **Subunit/Tissue** | **Breast** | **Umbilical Vein** | **Bone** | **Colon** | **Liver** | **Embryo** | **Skin** | **None** | **Brain** | **Cervix** | **Embryonic Kidney** | **Bone marrow** | **Blood** | **Lung** | **Mammary Gland** |
| --- | --- | --- | --- | --- | --- | --- | --- | --- | --- | --- | --- | --- | --- | --- | --- |
| **Core** | *SMARCA4* | *SMARCA4* | *SMARCA4* | *SMARCA4* | *SMARCA4* | *SMARCA4* | *SMARCA4* | *SMARCA4* | *SMARCA4* | *SMARCA4* | *SMARCA4* | *SMARCA4* | *SMARCA4* | *SMARCA4* | *SMARCA4* |
|  | *SMARCA2* | *SMARCA2* | *SMARCA2* | *SMARCA2* | *-* | *SMARCA2* | *SMARCA2* | *SMARCA2* | *SMARCA2* | *SMARCA2* | *SMARCA2* | *SMARCA2* | *SMARCA2* | *SMARCA2* | *SMARCA2* |
|  | *-* | *-* | *-* | *-* | *-* | *-* | *-* | *-* | *-* | *-* | *SMARCB1* | *-* | *-* | *-* | *-* |
|  | *SMARCC1* | *SMARCC1* | *SMARCC1* | *SMARCC1* | *SMARCC1* | *-* | *SMARCC1* | *SMARCC1* | *SMARCC1* | *SMARCC1* | *SMARCC1* | *SMARCC1* | *SMARCC1* | *SMARCC1* | *SMARCC1* |
|  | *SMARCC2* | *SMARCC2* | *SMARCC2* | *SMARCC2* | *SMARCC2* | *SMARCC2* | *SMARCC2* | *SMARCC2* | *SMARCC2* | *SMARCC2* | *SMARCC2* | *SMARCC2* | *SMARCC2* | *SMARCC2* | *SMARCC2* |
| **Accessory** | *-* | *-* | *ACTB* | *-* | *-* | *ACTB* | *-* | *ACTB* | *ACTB* | *ACTB* | *ACTB* | *ACTB* | *ACTB* | *ACTB* | *ACTB* |
|  | *-* | *-* | *-* | *ACTL6A* | *-* | *-* | *ACTL6A* | *-* | *ACTL6A* | *ACTL6A* | *ACTL6A* | *ACTL6A* | *ACTL6A* | *ACTL6A* | *ACTL6A* |
|  | *-* | *-* | *-* | *-* | *-* | *-* | *-* | *-* | *-* | *-* | *ACTL6B* | *ACTL6B* | *-* | *ACTL6B* | *-* |
|  | *SMARCD1* |  | *SMARCD1* | *SMARCD1* | *SMARCD1* | *SMARCD1* | *SMARCD1* | *SMARCD1* | *-* | *SMARCD1* | *SMARCD1* | *SMARCD1* | *SMARCD1* | *SMARCD1* | *SMARCD1* |
|  | *SMARCD2* | *SMARCD2* | *SMARCD2* | *SMARCD2* | *SMARCD2* | *SMARCD2* | *SMARCD2* | *SMARCD2* | *SMARCD2* | *SMARCD2* | *SMARCD2* | *SMARCD2* | *SMARCD2* | *SMARCD2* | *SMARCD2* |
|  | *-* | *-* | *SMARCD3* | *SMARCD3* | *-* | *SMARCD3* | *SMARCD3* | *-* | *SMARCD3* | *SMARCD3* | *SMARCD3* | *SMARCD3* | *SMARCD3* | *SMARCD3* | *SMARCD3* |
|  | *-* | *SMARCE1* | *SMARCE1* | *SMARCE1* | *SMARCE1* | *SMARCE1* | *-* | *-* | *SMARCE1* | *-* | *SMARCE1* | *SMARCE1* | *SMARCE1* | *SMARCE1* | *SMARCE1* |
| **Signature** | *ARID1A* | *ARID1A* | *ARID1A* | *ARID1A* | *ARID1A* | *ARID1A* | *ARID1A* | *ARID1A* | *ARID1A* | *ARID1A* | *ARID1A* | *ARID1A* | *ARID1A* | *ARID1A* | *ARID1A* |
|  |  | *ARID1B* | *ARID1B* | *-* | *ARID1B* | *ARID1B* |  | *ARID1B* | *ARID1B* | *ARID1B* | *ARID1B* | *ARID1B* | *ARID1B* | *ARID1B* | *ARID1B* |
|  | *ARID2* | *ARID2* | *ARID2* | *-* | *ARID2* | *ARID2* | *ARID2* | *ARID2* | *ARID2* | *ARID2* | *ARID2* | *ARID2* | *ARID2* | *ARID2* | *ARID2* |
|  |  |  | *BRD7* | *-* | *BRD7* | *BRD7* | *-* | *BRD7* | *BRD7* | *BRD7* | *-* | *BRD7* | *BRD7* | *BRD7* | *BRD7* |
|  | *BRD9* | *BRD9* | *BRD9* | *BRD9* | *BRD9* | *BRD9* | *BRD9* | *BRD9* | *BRD9* | *BRD9* | *BRD9* | *BRD9* | *BRD9* | *BRD9* | *BRD9* |
|  | *-* | *-* | *PHF10* | *-* | *PHF10* | *PHF10* | *PHF10* | *PHF10* | *PHF10* | *PHF10* | *PHF10* | *PHF10* | *PHF10* | *PHF10* | *PHF10* |
|  | *DPF1* | *-* | *-* | *DPF1* | *DPF1* | *DPF1* | *DPF1* | *DPF1* | *DPF1* | *DPF1* | *DPF1* | *DPF1* | *DPF1* | *DPF1* | *DPF1* |
|  | *-* | *-* | *-* | *-* | *-* | *-* | *DPF3* | *-* | *-* | *DPF3* | *-* | *DPF3* | *-* | *-* | *DPF3* |
|  | *-* | *DPF2* | *-* | *DPF2* | *-* | *-* | *-* | *DPF2* | *-* | *DPF2* | *DPF2* | *DPF2* | *DPF2* | *DPF2* | *DPF2* |
| **BAF Specific** | *-* | *BCL7A* | *-* | *-* | *-* | *-* | *BCL7A* | *-* | *-* | *BCL7A* | *BCL7A* | *BCL7A* | *BCL7A* | *BCL7A* | *BCL7A* |
|  | *-* | *BCL7B* | *BCL7B* | *BCL7B* | *BCL7B* | *BCL7B* | *BCL7B* | *BCL7B* | *BCL7B* | *BCL7B* | *BCL7B* | *BCL7B* | *BCL7B* | *BCL7B* | *BCL7B* |
|  | *BCL7C* | *BCL7C* | *BCL7C* | *BCL7C* | *BCL7C* | *BCL7C* | *BCL7C* | *BCL7C* | *-* | *BCL7C* | *BCL7C* | *BCL7C* | *BCL7C* | *BCL7C* | *BCL7C* |
|  | *-* | *-* | *-* | *-* | *-* | *-* | *-* | *-* | *-* | *-* | *-* | *-* | *BCL11A* | *BCL11A* | *BCL11A* |
|  | *-* | *BCL11B* | *-* | *BCL11B* | *-* | *-* | *BCL11B* | *-* | *BCL11B* | *-* | *BCL11B* | *-* | *BCL11B* | *BCL11B* | *BCL11B* |
|  | *-* | *SS18* | *-* | *SS18* | *SS18* | *-* | *-* | *SS18* | *SS18* | *SS18* | *SS18* | *SS18* | *SS18* | *SS18* | *SS18* |
|  | *-* | *-* | *-* | *-* | *SS18L1* | *-* | *-* | *-* | *-* | *-* | *-* | *SS18L1* | *SS18L1* | *SS18L1* | *SS18L1* |
| **PBAF Specific** | *-* | *PBRM1* | *-* | *-* | *-* | *-* | *-* | *PBRM1* | *-* | *PBRM1* | *PBRM1* | *PBRM1* | *PBRM1* | *PBRM1* | *PBRM1* |

**Supplementary table 3**: SWI/SNF subunit genes which have MYC binding peaks at the promoter regions identified using TFmapper across various tissues in human

| **Type of Subunit** | **Gene Names** | **E-box motifs and Myc binding peaks in MEF** | | | |
| --- | --- | --- | --- | --- | --- |
|  |  | **(-1000 to -500)** | **(-500 to -100)** | **(-100 to 0)** | **(0 to 100)** |
| CORE | SMARCA4 | YP | YP | P | P |
|  | SMARCA2 | P | YP | YP | P |
|  | SMARCB1 | YP | YP | YP | P |
|  | SMARCC1 | YP | YP | YP | YP |
|  | SMARCC2 | Y |  |  |  |
| Accessory | ACTB | YP | YP | P | P |
|  | ACTL6A | YP | YP | P | P |
|  | ACTL6B | Y | Y | Y |  |
|  | SMARCD1 | YP | YP | P | P |
|  | SMARCD2 | YP | P | YP | P |
|  | SMARCD3 | YP | YP | P | P |
|  | SMARCE1 | YP | YP | P | P |
| Signature | ARID1A | YP | YP | Y |  |
|  | ARID1B | Y |  |  |  |
|  | ARID2 | P | YP | P | P |
|  | BRD7 | Y | Y |  |  |
|  | BRD9 | Y | YP | P | P |
|  | PHF10 | Y | Y | Y |  |
|  | DPF1 | Y |  |  |  |
|  | DPF3 | YP | YP | P | P |
|  | DPF2 | Y | P | P | P |
| BAF Specific | BCL7A | Y | YP | YP | P |
|  | BCL7B | YP | YP | P | P |
|  | BCL7C | YP | YP | P | P |
|  | BCL11A | P | Y |  |  |
|  | BCL11B | YP |  | Y |  |
|  | SS18 | YP | P | P | P |
|  | SS18L1 | YP | P | P | P |
|  | BICRA (GLTSCR1) | Y |  |  |  |
| PBAF specific | PBRM1 | Y | YP | P | P |

**Supplementary table 4:** Position of E-box motifs and the MYC binding peaks in promoter region of SWI/SNF subunits in MEF

| **ABCE1** | 0.24 | **0.45** | **0.34** | **0.32** | **0.45** | **0.32** | 0.20 | **0.36** | **0.25** | **0.47** | 0.24 | **0.43** | **0.39** | **0.38** | 0.24 | **0.41** | **0.39** | **0.52** | **0.47** | **0.35** | **-0.32** | **0.58** | **0.37** | 0.26 | 0.27 |
| --- | --- | --- | --- | --- | --- | --- | --- | --- | --- | --- | --- | --- | --- | --- | --- | --- | --- | --- | --- | --- | --- | --- | --- | --- | --- |
| **WDR3** | **0.55** | **0.55** | **0.44** | **0.36** | **0.44** | **0.59** | **0.31** | **0.61** | **0.40** | **0.30** | 0.27 | **0.45** | **0.38** | 0.27 | **0.35** | **0.42** | **0.55** | **0.49** | 0.27 | 0.41 | -0.02 | **0.47** | 0.26 | **0.49** | **0.38** |
| **SMARCA4** | 0.25 | **0.35** | -0.07 | 0.22 | 0.23 | 0.27 | 0.09 | 0.14 | **0.41** | **-0.30** | 0.07 | 0.03 | -0.04 | -0.13 | -0.06 | 0.04 | 0.19 | 0.18 | 0.27 | 0.23 | -0.13 | **0.47** | 0.12 | 0.16 | -0.16 |
| **SMARCA2** | 0.20 | 0.23 | -0.12 | -0.01 | -0.05 | 0.28 | -0.15 | 0.03 | -0.16 | -0.22 | 0.09 | -0.08 | 0.01 | 0.20 | 0.07 | -0.01 | 0.01 | 0.08 | 0.02 | -0.19 | -0.05 | -0.06 | -0.01 | -0.01 | 0.24 |
| **SMARCB1** | 0.15 | **0.31** | -0.07 | 0.12 | 0.05 | -0.25 | 0.14 | 0.20 | **0.33** | -0.04 | 0.20 | 0.09 | -0.04 | 0.00 | -0.23 | **-0.41** | -0.09 | 0.23 | 0.12 | 0.08 | -0.04 | 0.22 | 0.10 | 0.17 | -0.25 |
| **SMARCC1** | **0.34** | **0.40** | -0.15 | 0.21 | 0.15 | 0.05 | **0.33** | 0.15 | **0.49** | 0.03 | -0.04 | 0.23 | 0.18 | -0.21 | 0.04 | 0.19 | **0.39** | 0.01 | 0.26 | 0.24 | 0.00 | **0.48** | 0.28 | **0.30** | **0.46** |
| **SMARCC2** | **-0.42** | 0.23 | **-0.40** | -0.11 | -0.23 | 0.13 | -0.01 | 0.00 | -0.06 | -0.22 | 0.09 | -0.16 | -0.02 | -0.05 | 0.01 | -0.34 | 0.01 | -0.15 | -0.08 | 0.01 | 0.08 | 0.19 | -0.03 | 0.04 | 0.07 |
| **ACTB** | -0.10 | **-0.38** | **0.39** | 0.02 | 0.21 | 0.01 | -0.18 | **-0.60** | 0.28 | 0.12 | 0.03 | 0.14 | 0.05 | -0.06 | -0.19 | 0.21 | -0.21 | -0.09 | -0.19 | -0.05 | **0.36** | 0.14 | **0.29** | -0.07 | **-0.32** |
| **ACTL6A** | 0.27 | **0.30** | 0.13 | 0.11 | 0.08 | 0.09 | 0.19 | **0.33** | 0.15 | 0.03 | 0.20 | 0.10 | 0.13 | 0.11 | -0.07 | 0.13 | 0.20 | 0.23 | 0.06 | 0.06 | -0.16 | **0.47** | **0.32** | 0.28 | **0.44** |
| **ACTL6B** | 0.00 |  | -0.12 | 0.06 | -0.05 | **-0.45** | -0.05 | 0.02 | -0.04 | -0.06 | 0.05 | -0.09 | 0.06 | -0.22 | -0.14 | -0.13 | -0.05 | -0.15 | -0.13 | 0.00 | 0.19 | -0.06 | -0.02 | -0.06 | -0.15 |
| **SMARCD1** | 0.27 | 0.27 | -0.01 | 0.04 | 0.13 | 0.13 | 0.03 | 0.01 | 0.20 | 0.10 | 0.12 | 0.00 | -0.05 | 0.10 | -0.27 | 0.02 | 0.05 | 0.21 | 0.01 | 0.20 | -0.03 | **0.29** | -0.03 | 0.11 | -0.16 |
| **SMARCD2** | 0.17 | 0.27 | -0.23 | -0.02 | -0.10 | -0.10 | **0.32** | -0.10 | -0.03 | -0.06 | 0.03 | 0.00 | 0.04 | -0.16 | 0.05 | 0.21 | 0.10 | 0.21 | 0.11 | 0.19 | 0.11 | **0.50** | 0.00 | 0.19 | -0.18 |
| **SMARCD3** | **-0.36** | **-0.38** | -0.23 | 0.09 | -0.24 | 0.15 | -0.16 | -0.26 | **-0.34** | -0.25 | -0.16 | -0.08 | -0.18 | -0.27 | 0.12 | -0.12 | -0.21 | -0.23 | -0.18 | -0.25 | **0.29** | **-0.43** | -0.24 | -0.07 | **-0.40** |
| **SMARCE1** | 0.02 | 0.06 | **-0.37** | -0.07 | -0.13 | 0.05 | 0.04 | 0.18 | 0.15 | -0.18 | 0.05 | 0.00 | -0.05 | 0.06 | -0.19 | 0.00 | -0.06 | -0.09 | 0.02 | 0.13 | **0.29** | -0.05 | 0.00 | -0.07 | **0.36** |
| **ARID1A** | **0.36** | 0.09 | -0.18 | -0.03 | 0.04 | -0.03 | -0.10 | 0.06 | 0.21 | 0.03 | **0.32** | -0.07 | -0.08 | 0.05 | -0.14 | 0.06 | **0.29** | 0.08 | 0.04 | -0.11 | -0.10 | **0.34** | 0.08 | 0.21 | 0.04 |
| **ARID1B** | 0.28 | **0.35** | -0.10 | -0.04 | -0.14 | 0.12 | 0.02 | 0.25 | 0.28 | -0.05 | 0.22 | 0.07 | 0.01 | 0.13 | 0.04 | 0.10 | 0.16 | 0.04 | **0.30** | -0.06 | 0.03 | 0.11 | 0.09 | 0.28 | 0.22 |
| **ARID2** | **-0.47** | 0.02 | **-0.33** | -0.23 | -0.16 | -0.02 | -0.06 | 0.07 | 0.05 | -0.05 | 0.17 | -0.08 | -0.08 | 0.04 | -0.13 | -0.03 | 0.13 | -0.19 | 0.08 | 0.01 | -0.21 | 0.24 | -0.02 | 0.02 | 0.29 |
| **BRD7** | **-0.25** | -0.01 | 0.01 | 0.04 | 0.21 | -0.08 | 0.00 | 0.18 | -0.05 | 0.11 | -0.09 | 0.03 | -0.08 | 0.04 | -0.02 | -0.06 | 0.05 | -0.12 | -0.03 | 0.05 | 0.15 | -0.18 | -0.14 | -0.01 | 0.29 |
| **BRD9** | -0.01 | 0.12 | -0.04 | 0.20 | 0.09 | -0.26 | -0.05 | 0.30 | 0.28 | 0.03 | 0.03 | 0.07 | 0.03 | -0.17 | -0.08 | -0.03 | 0.05 | -0.01 | -0.11 | -0.16 | 0.20 | -0.07 | 0.03 | 0.04 | -0.14 |
| **PHF10** | -0.17 | 0.20 | -0.17 | 0.06 | -0.19 | -0.06 | 0.02 | 0.20 | -0.03 | **-0.31** | 0.19 | -0.10 | -0.05 | -0.13 | -0.04 | -0.06 | 0.06 | -0.27 | -0.11 | -0.03 | 0.10 | -0.13 | 0.13 | 0.12 | 0.22 |
| **DPF1** | 0.20 | 0.16 | 0.18 | 0.07 | 0.18 | 0.19 | 0.11 | 0.19 | 0.03 | 0.28 | 0.12 | -0.02 | 0.04 | -0.23 | -0.21 | -0.05 | 0.10 | -0.13 | -0.09 | 0.12 | -0.05 | **0.36** | 0.12 | 0.03 | -0.15 |
| **DPF2** | 0.14 | **0.43** | 0.01 | 0.05 | -0.01 | -0.03 | 0.16 | -0.04 | 0.04 | -0.04 | 0.05 | 0.09 | -0.09 | -0.21 | -0.09 | -0.01 | -0.12 | -0.11 | 0.00 | 0.11 | -0.14 | -0.02 | -0.07 | 0.19 | -0.07 |
| **DPF3** | -0.06 | -0.09 | 0.23 | 0.07 | 0.06 | 0.22 | 0.02 | -0.22 | -0.06 | -0.04 | 0.10 | -0.14 | 0.04 | 0.05 | 0.12 | 0.15 | 0.05 | **0.29** | -0.24 | -0.05 | -0.13 | 0.27 | 0.04 | 0.12 | 0.06 |
| **BCL7A** | 0.17 | **0.34** | -0.18 | 0.10 | 0.05 | -0.17 | 0.26 | -0.14 | 0.24 | 0.10 | -0.15 | -0.02 | 0.02 | 0.06 | -0.21 | -0.13 | 0.18 | -0.08 | **0.33** | 0.13 | **-0.51** | **0.51** | 0.08 | 0.22 | **0.40** |
| **BCL7B** | -0.18 | **-0.30** | -0.02 | -0.03 | 0.02 | 0.13 | -0.15 | -0.20 | -0.28 | 0.06 | -0.08 | -0.08 | 0.08 | -0.12 | -0.09 | -0.17 | **-0.32** | -0.26 | **-0.38** | -0.04 | -0.16 | -0.04 | -0.07 | -0.19 | **-0.47** |
| **BCL7C** | -0.22 | 0.06 | -0.19 | 0.00 | -0.15 | -0.18 | 0.02 | -0.28 | -0.14 | -0.20 | -0.07 | -0.19 | -0.15 | **-0.43** | -0.08 | -0.04 | **-0.33** | -0.24 | -0.08 | -0.07 | 0.05 | -0.26 | -0.16 | -0.30 | **-0.34** |
| **BCL11A** | 0.09 | 0.42 | 0.13 | **0.41** | 0.20 | -0.14 | **0.31** | **0.32** | 0.22 | -0.19 | 0.23 | -0.02 | -0.02 | 0.01 | 0.12 | -0.02 | -0.10 | 0.12 | -0.08 | -0.02 | **0.62** | 0.00 | 0.01 | 0.10 | **0.42** |
| **BCL11B** | 0.20 | -0.02 | 0.28 | 0.21 | **0.34** | **0.36** | 0.23 | -0.18 | 0.14 | 0.08 | 0.30 | 0.22 | 0.09 | 0.10 | -0.08 | **0.35** | -0.11 | -0.03 | -0.27 | 0.08 | **0.41** | 0.21 | 0.16 | 0.04 | -0.26 |
| **SS18** | **0.30** | 0.12 | 0.08 | 0.08 | 0.08 | -0.02 | **-0.39** | 0.11 | -0.08 | -0.10 | -0.14 | -0.09 | -0.09 | 0.01 | -0.16 | 0.02 | -0.26 | -0.08 | -0.22 | -0.09 | **-0.43** | **-0.30** | 0.13 | 0.00 | 0.05 |
| **SS18L1** | -0.14 | 0.26 | -0.37 | 0.04 | -0.28 | -0.27 | **0.32** | 0.14 | 0.15 | -0.05 | 0.05 | -0.09 | -0.16 | -0.10 | -0.13 | -0.22 | 0.21 | -0.13 | 0.18 | -0.02 | **-0.35** | 0.03 | 0.01 | 0.03 | 0.14 |
| **BICRA** | -0.04 | -0.12 | -0.32 | -0.02 | -0.07 | -0.24 | 0.06 | 0.16 | **0.36** | -0.16 | 0.19 | -0.06 | -0.11 | 0.00 | -0.05 | -0.10 | -0.11 | 0.15 | 0.04 | -0.10 | -0.16 | -0.03 | -0.17 | -0.21 | -0.26 |
| **PBRM11** | 0.06 | -0.06 | -0.27 | -0.06 | -0.14 | -0.02 | -0.02 | 0.02 | **0.36** | -0.05 | 0.07 | -0.02 | 0.02 | -0.05 | -0.05 | 0.09 | 0.16 | -0.10 | 0.21 | -0.04 | 0.08 | 0.23 | 0.25 | 0.12 | **0.39** |
| **SWI/SNF Subunit /Control Gene** | Adrenocortical Carcinoma (92) | Acute Myeloid Leukemia (200) | Bladder Urothelial Carcinoma (411) | Breast Invasive Carcinoma (1084) | Cervical Squamous Cell Carcinoma (297) | Cholangiocarcinoma (36) | Colorectal Adenocarcinoma (526) | Diffuse Large B-Cell Lymphoma (48) | Glioblastoma Multiforme (592) | Head and Neck Squamous Cell Carcinoma (523) | Kidney Renal Clear Cell Carcinoma (512) | Liver Hepatocellular Carcinoma (372) | Lung Adenocarcinoma (566) | Mesothelioma (87) | Ovarian Serous Cystadenocarcinoma (585) | Pancreatic Adenocarcinoma (184) | Prostate Adenocarcinoma (494) | Sarcoma(255) | Skin Cutaneous Melanoma(448) | Stomach Adenocarcinoma (440) | Testicular Germ Cell Tumors (149) | Thymoma (123) | Thyroid Carcinoma(500 ) | Uterine Corpus Endometrial Carcinoma(529) | Uveal Melanoma (80) |

**Supplementary table 5**: Co-expression correlation of MYC and SWI/SNF subunit genes in the panel of cancer cancers available in the TCGA PanCancerAtlas through cBioPortal. The Pearson correlation coefficient is reported and the significant correlation coefficient with value >0.3 and p-value<0.05 are highlighted. The number of samples in each cancer type available in TCGA PanCancerAtlas is reported in brackets.

| **SWI/SNF Subunit/Control Gene name** | **MEF (GSE67715)** | | **MEF (GSE102917)** | | **TGR1-HO15 (GSE18845)** | | **Medulloblastoma (GSE22139)** | |
| --- | --- | --- | --- | --- | --- | --- | --- | --- |
|  | **Log FC** | **P-value** | **Log FC** | **Adj.P.val** | **Log FC** | **Adj.P.val** | **logFC** | **Adj. P.val** |
| **SMARCA4** | 0.14 | 0.55 | **3.76** | **0** | -0.05 | 6.80E-01 | -0.22 | 0.174076991 |
| **SMARCA2** | -0.05 | 1 | **-0.61** | **0.004** |  |  | **-0.79** | **2.13E-40** |
| **SMARCB1** | -0.02 | 0.94 | **-3.75** | **3.69E-87** | 0.11 | 0.378 | -0.05 | 3.95E-01 |
| **SMARCC1** | -0.28 | 0.42 | **1.19** | **5.65E-11** | **-0.37** | **0.000922** | **0.65** | **8.48E-27** |
| **SMARCC2** | 0.22 | 0.43 | **2.69** | **1.28E-24** |  |  | -0.08 | 0.83684186 |
| **ACTB** | -0.08 | 0.02 | **-1.54** | **1.61E-17** | 0.16 | 0.118 | -0.36 | **5.93E-156** |
| **ACTL6A** | -0.09 | 0.84 | **-2.79** | **8.35E-39** | **-0.28** | **0.0305** | 0.09 | 0.270753785 |
| **ACTL6B** | 0.21 | 1 | **-2.97** | **1.77E-77** | 0.06 | 0.715 | -0.01 | 1 |
| **SMARCD1** | 0.15 | 0.74 | **1.09** | **0.0000539** | -0.15 | 0.209 | 0.05 | 0.705479953 |
| **SMARCD2** | 0.02 | 1 | **5.99** | **1.82E-31** | 0.03 | 0.9 | 0.43 | **2.46497E-09** |
| **SMARCD3** | -0.23 | 0.59 | **-2.3** | **2.74E-17** | 0.15 | 0.284 | **0.55** | **1.37E-41** |
| **SMARCE1** | 0.02 | 1 | **2.4** | **0.00000164** | -0.1 | 0.442 | -0.19 | 0.000251734 |
| **ARID1A** | -0.03 | 1 | **-4.6** | **1.16E-23** | -0.15 | 0.327 | -0.12 | 0.407256918 |
| **ARID1B** | 0.06 | 1 | **-0.72** | **0.064587197** |  |  | -0.14 | 0.3953887 |
| **ARID2** | 0.06 | 1 | **1.66** | **0.026820284** | 0.11 | 0.41861 | 0.24 | 0.271065337 |
| **BRD7** | 0.17 | 0.56 | **1.99** | **6.35E-11** | -0.16 | 0.13 | 0.05 | 0.407256918 |
| **BRD9** | 0.05 | 1 | **0.97** | **0.00000635** | -0.016 | 0.937 | 0.33 | **2.99E-07** |
| **PHF10** | -0.5 | 0.08 | **-3.1** | **3.33E-61** | -0.04 | 0.791 | 0.14 | 0.002695382 |
| **DPF1** | -0.44 | 0.75 | -0.07 | 0.64 | -0.02 | 0.887 | -0.12 | 0.407256918 |
| **DPF3** | 0.05 | 1 | **-1.74** | **7.36E-17** | -0.017 | 0.926 | -0.25 | 0.270753785 |
| **DPF2** | 0.01 | 1 | **-0.57** | **0.00000107** | -0.11 | 0.41 | -0.12 | **0.023683384** |
| **BCL7A** | -0.42 | 0.77 | **-2.23** | **2.13E-11** | -0.16 | 0.244447 | **0.75** | **1.36E-24** |
| **BCL7B** | 0.25 | 0.53 | **-1.6** | **1.48E-14** | **0.25** | **0.0451** | 0.46 | **4.58E-17** |
| **BCL7C** | -0.35 | 0.55 | **-1.14** | **0.00000203** | -0.25 | 0.223 | **-0.48** | **3.83E-14** |
| **BCL11A** | -0.35 | 1 | **-1.56** | **5.28E-14** | -0.06 | 0.674 | **0.74** | **2.41E-47** |
| **BCL11B** | 0.09 | 1 | **-1.26** | **8.27E-12** | 0.008 | 1 | **0.77** | **2.21E-18** |
| **SS18** | 0.21 | 0.38 | **-4.33** | **0** | -0.06 | 0.733 | **-0.74** | **1.18E-41** |
| **SS18L1** | 0.24 | 1 | **-5.54** | **0** | 0.03 | 0.86 | 0.12 | 0.83684186 |
| **BICRA (GLTSCR1)** | -0.11 | 1 | 0.25 | 1 | -0.02 | 0.886 | -0.38 | **1.46E-06** |
| **PBRM1** | 0.32 | 0.45 | **-0.55** | **4.33E-05** |  |  | -0.45 | **1.31E-12** |
| **WDR3** | -0.44 | 0.32 | **1.53** | **3.09E-07** | **-0.72** | **0.0000492** | **0.71** | **0.045443285** |
| **ABCE1** | **-1.16** | **6.12E-08** | **2.53** | **1.72E-28** | **-0.53** | **0.0000298** | **0.55** | **1.06E-40** |

**Supplementary table 6**: Results from the differential expression analysis in MYC perturbed conditions in MEFs, MYC proficient and deficient cell lines, TGR1 and HO15 and Medulloblastoma. Fold change >|0.5| with significant Adj. P-value are highlighted in bold. The values for the subunit genes whose expression details are not available are kept blank.

| **Type of Subunit** | **Subunit Gene Name** | **Regulation by MYC** | **Cell types showing regulation** |
| --- | --- | --- | --- |
| CORE | SMARCA4 | Positive/Negative | **Acute Myeloid Leukemia, Glioblastoma Multiforme,** Head and Neck Squamous Cell Carcinoma**, Thymoma** |
|  | SMARCA2 | Negative | Medulloblastoma |
|  | SMARCB1 | Positive | **Acute Myeloid Leukemia, Glioblastoma Multiforme** |
|  | SMARCC1 | Positive | **Medulloblastoma, Adrenocortical Carcinoma, Acute Myeloid Leukemia, Colorectal Adenocarcinoma, Glioblastoma Multiforme, Prostate Adenocarcinoma, Thymoma, Uterine Corpus Endometrial Carcinoma, Uveal Melanoma** |
|  | SMARCC2 | Negative | Adrenocortical Carcinoma, Bladder Urothelial Carcinoma, Pancreatic Adenocarcinoma |
| Accessory | ACTB | Positive/Negative | Acute Myeloid Leukemia, **Bladder Urothelial Carcinoma**, Diffuse Large B-Cell Lymphoma, **Testicular Germ Cell Tumors**, Uveal Melanoma |
|  | ACTL6A | Positive | **Acute Myeloid Leukemia, Diffuse Large B-Cell Lymphoma, Thymoma, Thyroid Carcinoma, Uveal Melanoma** |
|  | ACTL6B | Negative | Medulloblastoma, Cholangiocarcinoma |
|  | SMARCD1 | Positive | **Thymoma** |
|  | SMARCD2 | Positive | **Colorectal Adenocarcinoma**, **Thymoma** |
|  | SMARCD3 | Negative | Adrenocortical Carcinoma, Acute Myeloid Leukemia, Glioblastoma Multiforme, Thymoma**,** Uveal Melanoma |
|  | SMARCE1 | Positive/Negative | Medulloblastoma, Bladder Urothelial Carcinoma, **Uveal Melanoma** |
| Signature | ARID1A | Positive | **Adrenocortical Carcinoma, Kidney Renal Clear Cell Carcinoma, Thymoma** |
|  | ARID1B | Positive | **Acute Myeloid Leukemia, Skin Cutaneous Melanoma** |
|  | ARID2 | Positive/Negative | Medulloblastoma, **Mouse Embryonic Fibroblast,** Adrenocortical Carcinoma, Bladder Urothelial Carcinoma |
|  | BRD7 | Negative | Medulloblastoma |
|  | BRD9 | - | - |
|  | PHF10 | Positive/Negative | **Mouse Embryonic Fibroblast,** Head and Neck Squamous Cell Carcinoma |
|  | DPF1 | Positive | **Thymoma** |
|  | DPF2 | Positive | **Mouse Embryonic Fibroblast, Acute Myeloid Leukemia** |
|  | DPF3 | - | - |
| BAF Specific | BCL7A | Positive/Negative | Medulloblastoma**, Acute Myeloid Leukemia, Skin Cutaneous Melanoma,** Testicular Germ Cell Tumors**, Thymoma, Uveal Melanoma** |
|  | BCL7B | Negative | Acute Myeloid Leukemia, Prostate Adenocarcinoma, Skin Cutaneous Melanoma, Uveal Melanoma |
|  | BCL7C | Negative | Medulloblastoma, Mesothelioma, Prostate Adenocarcinoma, Uveal Melanoma |
|  | BCL11A | Positive | **Medulloblastoma, Acute Myeloid Leukemia, Breast Invasive Carcinoma, Colorectal Adenocarcinoma, Diffuse Large B-Cell Lymphoma, Testicular Germ Cell Tumors, Uveal Melanoma** |
|  | BCL11B | Positive | **Medulloblastoma, Cervical Squamous Cell Carcinoma, Cholangiocarcinoma, Pancreatic Adenocarcinoma, Testicular Germ Cell Tumors** |
|  | SS18 | Positive/Negative | Medulloblastoma**, Adrenocortical Carcinoma**, Colorectal Adenocarcinoma, Testicular Germ Cell Tumors, Thymoma |
|  | SS18L1 | Positive/Negative | Bladder Urothelial Carcinoma, **Colorectal Adenocarcinoma**, Testicular Germ Cell Tumors |
|  | BICRA (GLTSCR1) | Positive/Negative | Bladder Urothelial Carcinoma, **Glioblastoma Multiforme**, |
| PBAF specific | PBRM1 | Positive | **Glioblastoma Multiforme, Uveal Melanoma** |

**Supplementary Table 7:** Summary of SWI/SNF subunit regulation by MYC. Cell types showing positive regulation are highlighted in bold.

| **S. No.** | **Organism** | **Cell Type** | **GEO ID** | **GEO samples used for the analysis** | **Experiment** | **Analysis performed in the present study** |
| --- | --- | --- | --- | --- | --- | --- |
| 1 | Mouse | MEF | GSE67715 | GSM1654749 GSM1654750 GSM1654753 GSM1654754 | Expression profiling by high-throughput sequencing with control and Myc siRNA in MEF | Differential expression analysis(Results included in supplementary table 6) |
| 2 | Mouse | MEF | GSE102917 | GSM2748251  GSM2748252 | Genome-wide transcriptome profile in response to expression of ectopic MYC proteins [RNA-Seq] | Differential expression analysis(Results included in supplementary table 6) |
| 3 | Mouse | MEF | GSE63756 | GSM1556707  GSM1556708  GSM1556712  GSM1556713  GSM1556714  GSM1556718  GSM1556719  GSM1556722  GSM1556723  GSM1556726  GSM1556727  GSM1556731  GSM1556732  GSM1556736  GSM1556737  GSM1556738  GSM1556742  GSM1556743  GSM1556747  GSM1556748  GSM1556749  GSM1556753  GSM1556754  GSM1556758  GSM1556759  GSM1556760  GSM1556763  GSM1556764  GSM1556765  GSM1556769  GSM1556770  GSM1556771 | Expression profiling by high-throughput sequencing MEF across eight mouse strains | Expression levels of Myc and SWI/SNF subunits in MEF from different strains of mouse(Reported in Figure 3A) Correlation analysis between Myc and SWI/SNF subunits also performed based on this data. |
| 4 | Rat | TGR1, HO15 | GSE18845 | GSM467052 GSM467053 GSM467054 GSM467058 GSM467059 GSM467060 | Expression profiling by array from fibroblasts that are wild-type for c-Myc (TGR-1 cells) and null for c-Myc (HO15.19 cells) | Differential expression analysis in MYC proficient and deficient rat cells (Results included in supplementary table 6) |
| 5 | Human | medulloblastoma | GSE50765 | Gene expression profile available for 83 samples used | Affymetrix Human Gene 1.1 ST Array profiling of 83 primary SHH-driven medulloblastoma samples | Co-expression correlation analysis of SWI/SNF subunits with MYC. (Results reported in Figure 3C) |
| 6 | Human | Medulloblastoma | GSE22139 | GSM550698  GSM550699 GSM550700 GSM550701 GSM550702 GSM550703 | Gene expression profiling by array to compare the effects of overexpressing and silencing MYC on the transcriptome of a MB-derived cell line. | Differential expression analysis in MYC overexpressing and silenced Medulloblastoma Cells (Results included in supplementary table 6) |
| 7 | Mouse | MEF | GSE109458 | GSM3425926 | Genome wide binding profiling of MYC in MEF | MYC binding peaks at the promoter locus of SWI/SNF subunit genes in MEF were visualized (Figure 2B) |
| 8 | Human | MCF10A | GSE31477 | GSM935491 | Genome wide binding profiling by ENCODE | MYC binding peaks at the promoter locus of SWI/SNF subunit genes in MEF were visualized (Supplementary figure 1B) |

**Supplementary table 8**: Gene expression and MYC binding profile dataset used in the study.
